# 

**Published:** 1916-07

**Authors:** 


CONTENTS.

| | Page |
|---|---|
| The Reduction of Disabilities from Wounds in War. By W. GORDON, M.D., F.R.C.P. | 1 |
| On the Work of a Military General Hospital in Egypt. By ERNEST HEY GROVES, M.D., M.S., F.R.C.S. | 11 |
| Note on the Technique of Sphenoidal Sinus Exploration for Meningococcal and other Infections. By Major P. WATSON-WILLIAMS, R.A.M.C.(T.), M.D. Lond. | 21 |
| Experiences of an Ear, Nose and Throat Specialist at one of the Bases. By Captain J. P. I. HARTY, R.A.M.C.(T.), M.B., B.Ch., F.R.C.S. | 39 |
| Some Neuroses of the War. By Lieut.-Col. J. MICHELL CLARKE, R.A.M.C.(T.), M.A., M.D., LL.D., F.R.C.P. | 49 |
| Notes on the Enteric Group of Diseases as seen in an Isolation Hospital in France. By Captain J. M. FORTESCUE-BRICKDALE, R.A.M.C.(T.), M.A., M.D., M.R.C.P. | 72 |
| Forensic Examination of Blood-stains in the Tropics. By the late FRANCIS SHINGLETON SMITH, B.A., M.B., B.C. Cantab. | 82 |
| Reviews of Books | 93 |
| Editorial Notes | 100 |
| Meetings of Societies | 100 |
| Obituary Notices | 101 |

